# A Note on Why Low-Symmetry Lanthanide Clusters Can Be Good Single Molecule Magnets

**DOI:** 10.1021/jacs.5c16415

**Published:** 2025-12-24

**Authors:** Oliver Waldmann

**Affiliations:** Physikalisches Institut, Universität Freiburg, D-79104 Freiburg, Germany

## Abstract

Lanthanide complexes with a low-symmetry ligand environment are frequently observed to exhibit good single molecule magnet (SMM) behavior, which is seemingly at odds with the established understanding that strong Ising-type magnetic anisotropy is essential. This and other paradoxical features of such clusters are shown to follow naturally from a semiclassical treatment of the ligand field, which becomes valid in the limit of large total angular momentum *J*. Quantum corrections are discussed qualitatively. The resulting physical picture offers clear insight into a broad class of lanthanide-based SMMs.

## Graphical abstract

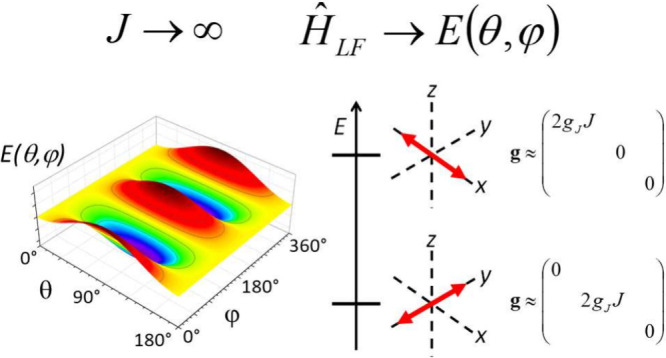

One of the intriguing and still unresolved mysteries in the field of lanthanide-based single molecule magnets (SMMs) is the empirical observation that lanthanide clusters with low-symmetry ligand environments can exhibit strong SMM behavior. This is in contrast to the established understanding, derived from studies on 3d-based SMMs, that strong Ising-type magnetic anisotropy is essential for the emergence of SMM phenomena.^1^ The blocking temperature for slow magnetic relaxation and quantum tunneling of the magnetization have been associated with the height of the anisotropy barrier for spin reversal and a relative absence of transversal terms in the spin Hamiltonian. The ligand field should hence be strongly axial (“axial” in the sense of a dominant *D* term in the spin Hamiltonian). The SMMs Mn_12_ and Fe_8_ are prototypical examples.^2–6^

This design approach has led to enormous success also for lanthanide-based SMMs in terms of increased blocking temperatures,^7–20^ but a number of lanthanide-based SMMs have been identified which appear to defeat this rule.^21–29^ Some characteristic features have empirically been collected:

(i) The ligand environment is of low symmetry or symmetry is absent.
(ii) The ground doublet is strongly axial; “axial” meaning here that the **g** matrix characterizing the doublet is nearly that of a doublet of M = ± *J* spin states with principal values *g*_*x*_ = *g*_*y*_ = 0 and *g*_*z*_ = 2*g*_*J*_ *J* (*M* is the magnetic quantum number, *g*_*J*_ is the Landé *g* factor, *J* > 1/2 is generally assumed). The orientation of the **g** matrix determines the orientation of the main magnetic anisotropy axis.^30,31^

(iii) In addition to a strongly axial doublet in the ground state another strongly axial doublet appears in the antiground state (states of highest energy), which is characterized by a **g** matrix with principal values very similar to those of the ground doublet but which is rotated compared to it.^23,24,32^

That is, noting the two senses of “axial”, the paradox is that a low-symmetry, nonaxial ligand field can lead to two strongly axial doublets, one in the ground and one in the antiground state.

Understanding the situation is not academic but of significant practical relevance. For instance, if a ligand field gives rise to strongly axial doublets in the ground and antiground states, then its inverse will do so too. It may then not be easily clear what the “sign” of the ligand field is, defeating proper understanding. The situation can typically arise, e.g., for oblate lanthanide ions in equatorial ligand environments such as in [Dy^III^DOTA­(H_2_O)­Na_3_]^2+^,^33,34^ or prolate lanthanide ions in low-symmetry environments.^8^

In this note, the physical mechanism giving rise to the above features is presented qualitatively. The main idea is a semiclassical treatment of the ligand field, or the approximation of a large total angular moment *J*, which for late lanthanides such as Dy^III^ seems reasonable. It will be shown that the above seemingly paradoxical features are quite naturally explained in the semiclassical limit *J* → *∞*. Explaining the deviation from these features is in fact the (theoretically) difficult part, and it is here where the discussion will remain qualitative.

The discussion directly applies to Kramers ions, where doublets consist of degenerate states and characterization by a **g** matrix is obvious.^35^ It can often be carried over also to non-Kramers ions when the notion of quasi-degenerate doublets is meaningful, e.g., when the (tunnel) splitting in the ground and antiground doublets are sufficiently small.^36^ In the following, the focus is on Kramers ions.

The lanthanide ion is described by the common ligand-field Hamiltonian in the space of the *J* multiplet, $Ĥ=\underset{kq}{\sum }B_{kq}{Ô}_{kq}$ , where ${Ô}_{kq}$ are Stevens operators,^37^ and *k* is restricted to even values and by *k* ≤ 2*J*.^35^ It will be convenient to also work with operators ${Ŷ}_{kq}$ which transform like the spherical harmonics *Y*_*kq*_;^36,38^ the ligand-field Hamiltonian then reads

1 $$Ĥ=\underset{kq}{\sum }C_{kq}{Ŷ}_{kq}$$

In the classical limit the spin operators become vectors,^39,40^ i.e.,

2 J⃗^→Jn⃗

where $\mathrm{n⃗}={\left(\cos\varphi \,\sin\theta ,\sin\varphi \,\sin\theta ,\cos\theta \right)}^{T}$ , with the polar and azimuth angles θ, φ. The ligand-field Hamiltonian then becomes a potential energy in spherical coordinates, which in the classical limit reads

3 $$Ĥ\to E^{cl}\left(\theta ,\varphi \right)=\underset{kq}{\sum }J^{k}C_{kq}Y_{kq}\left(\theta ,\varphi \right)$$

*E* ^*cl*^(θ, φ) will be denoted as classical energy (see also the Supporting Information (SI)).

The ground and antiground states of Ĥ are evidently related to the minimal and maximal energies of *E* ^*cl*^(θ, φ). Due to time-reversal symmetry, every minimum at n⃗ has a mirror image at −n⃗ , i.e., the number of minima is always even. The reasoning for the maxima mirrors that for the minima, except that the energies are inverted, and for brevity the maxima are typically not discussed explicitly in the following.

The semiclassical description is commonly based on spin coherent states,^41^ which are simply the quantum spin state |*J*, *J*⟩ rotated into the direction of a unit vector n⃗ : |θ,φ⟩=R̂(θ,φ)|J,J⟩ , where R̂(θ,φ) is the rotation operator associated with the Euler angles θ, φ. The state in direction −n⃗ can be associated with the time-reversed companion T̂|θ,φ⟩=R̂(θ,φ)|J,−J⟩ , where T̂ is the time-reversal operator. The pair of states |θ, φ⟩ and T̂|θ,φ⟩ thus form a doublet which is associated with the pair of directions n⃗ and −n⃗ .

It is straightforward to calculate the expectation value ⟨θ,φ|Ĥ|θ,φ⟩ ,^36,38^ which will be denoted as semiclassical energy *E* ^*sc*^(θ, φ). For *J* → *∞* it matches the classical energy *E* ^*cl*^(θ, φ) (the difference between *E* ^*sc*^ and *E* ^*cl*^ is irrelevant for a qualitative discussion, see also SI).

The key point to note is that the **g** matrix for the doublet of rotated spin states R̂(θ,φ)|J,±J⟩ is obviously maximally axial, and rotated into the same direction, i.e.,

4 $$g_{\left|\theta ,\varphi \right\rangle }=R^{-1}\left(\theta ,\varphi \right)\left(\begin{array}{lll} 0 & 0 & 0\\ 0 & 0 & 0\\ 0 & 0 & 2g_{J}J \end{array}\right)R\left(\theta ,\varphi \right)$$

where *R*(θ, φ) is the rotation matrix associated with the rotation operator R̂(θ,φ) .

In other words, the situation is simply that: A global minimum in the potential *E* ^*cl*^(θ, φ) at (θ_min_, φ_min_) implies a spin coherent state |θ_min_, φ_min_⟩, which in turn implies a ground doublet which is characterized by a maximally axial **g** matrix oriented in the direction of (θ_min_, φ_min_). This holds analogously for a global maximum.

The potential *E* ^*cl*^(θ, φ) always exhibits two global minima, but can additionally possess extra global or local minima. Usually, these are separated by barriers and occur at discrete points on the unit sphere, i.e., are isolated minima. However, they may also form closed, continuous lines corresponding to flat valleys. The simplest case is thus that of two global isolated minima at ${\mathrm{n⃗}}_{\min}$ and $-{\mathrm{n⃗}}_{\min}$ , which are separated by comparatively large barriers from each other as well as from additional local minima. This translates analogously to maxima.

The following examples are instructive.^39,42^ The operator ${Ô}_{20}=3{Ĵ}_{z}^{2}-J\left(J+1\right)$ becomes

5 $${Ô}_{20}\to O_{20}=J^{2}\left(3\cos\theta^{2}-1\right)$$

in the classical limit (see also SI) and ${Ô}_{22}={Ĵ}_{x}^{2}-{Ĵ}_{y}^{2}$ becomes

6 $${Ô}_{22}\to O_{22}=J^{2}\cos2\varphi \sin\theta^{2}$$

A third example is ${Ô}_{20}+\alpha {Ô}_{22}$ with α ≥ 0. The potentials *O*_20_, *O*_22_ and *O*_20_ + *αO*_22_ with α = 1/4 and α = 1 are shown in Figure 1.

**Figure 1:**
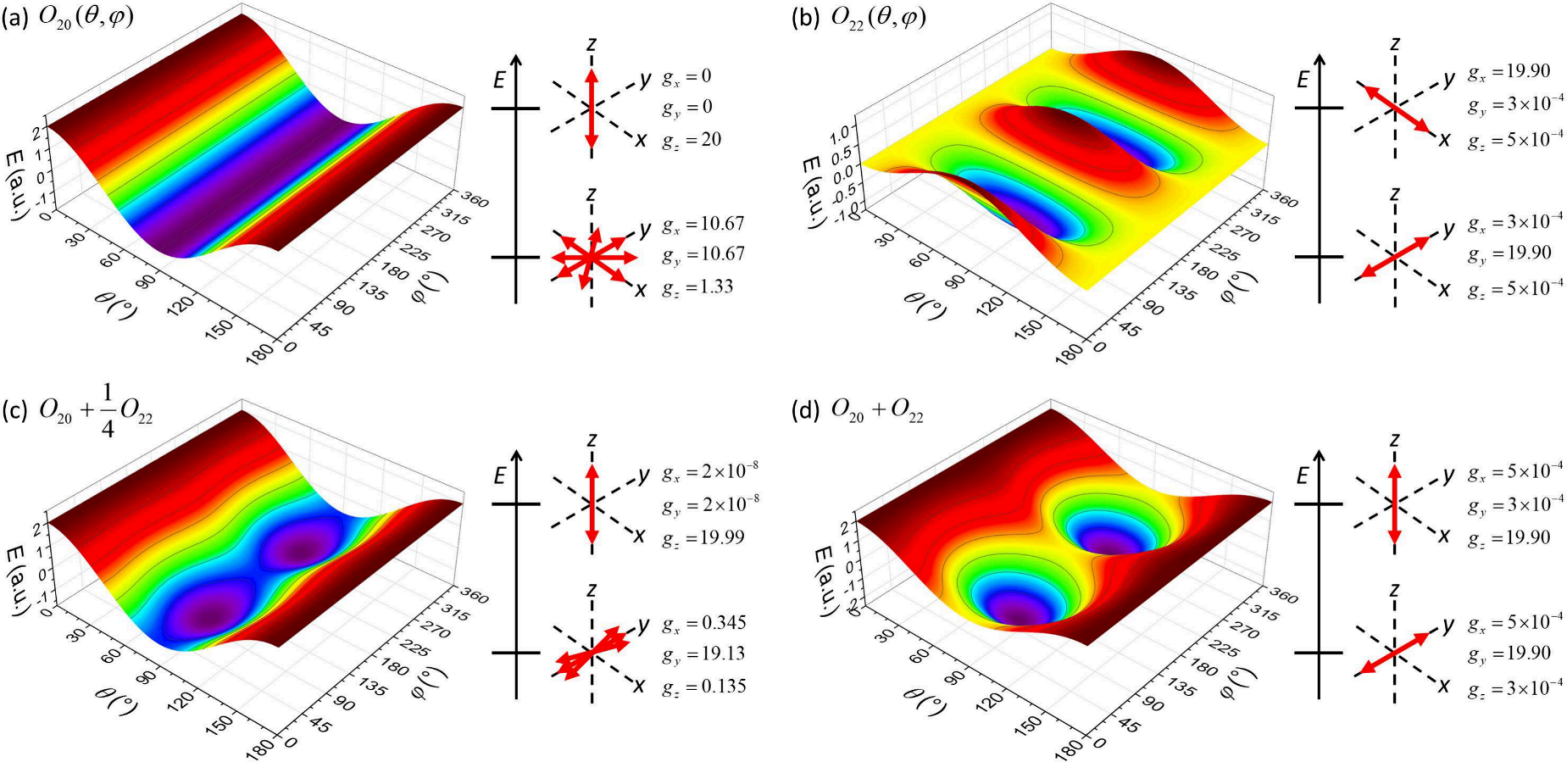
Color contour plots of the potentials corresponding to the operators (a) ${Ô}_{20}$ , (b) ${Ô}_{22}$ , and ${Ô}_{20}+\alpha {Ô}_{22}$ with (c) α = 1/4 and (d) α = 1. Also shown are illustrations of the orientation of the magnetic anisotropy axes in the ground and antiground doublets, and the magnitudes of the principal *g* values for Dy^III^ (2*g*_*J*_ *J* = 20).

These examples adequately demonstrate the relevant features. For α > 0, the model *O*_20_ + *αO*_22_ generically exhibits isolated global minima at (θ, φ) = (90°,90°) and (90°,270°), which are separated by large barriers at the north and south poles. They are also separated by barriers along the equator, but these are low for small α. For α = 0, they disappear entirely, and a flat minimum valley emerges, reflecting the axial symmetry in this case. The potential exhibits two isolated maxima at (θ, φ) = (0°,0°), (0°,180°) (north and south poles) for α < 3, whereas for α > 3 they appear at (θ, φ) = (90°,0°), (90°,180°). Thus, for α = 3 maximum ridges exist at φ = 0° and 180°.

The location of the extrema allows us to read off the anisotropy directions in the ground and antiground doublets. The potential *O*_22_ immediately shows that the operator ${Ô}_{22}$ exhibits a strongly axial ground doublet, with the anisotropy axis lying in the equatorial plane along the *y* direction. In addition, it exhibits a strongly axial antiground doublet with the anisotropy axis along the *x* direction. That is, strongly axial doublets appear in both the ground and antiground state with a relative orientation of their **g** matrices of 90° (see pictogram in Figure 1b). This is easily confirmed by explicitly diagonalizing ${Ô}_{22}$ (for large *J*), and reflects its chiral symmetry.^43^ Also for ${Ô}_{20}+\alpha {Ô}_{22}$ with α ≠ 0, 3 two strongly axial doublets with rotated **g** matrices are obtained (see pictogram in Figure 1d).

For ${Ô}_{20}$ the situation is more involved. *O*_20_ exhibits two isolated maxima at the poles, which are obviously associated with (nonrotated) |*J*, ± *J*⟩ spin states. Indeed, ${Ô}_{20}$ exhibits a maximally axial doublet in the antiground state with the anisotropy axis along the *z* direction (see pictogram in Figure 1a). The minimal energies in *O*_20_, however, form a flat valley, and the situation is less straightforward due to quantum corrections.

Quantum corrections can indeed modify the picture, and this is why the paradoxical features present in the classical analysis of eq 1 are not always observed. The basic aspects are well-known, e.g., from discussions of quantum phenomena in 3d-based SMMs. Quantum fluctuations, tunneling and interference effects associated with classical paths between potential wells lead to tunneling splittings (quenched for Kramers ions) and energy shifts, thereby adding a quantum contribution to the classical energy. Qualitatively, the corrections increase as barrier heights decrease (comprehensive treatments are available elsewhere^1,40,44–46^ ).

For the ${Ô}_{20}+\alpha {Ô}_{22}$ model, the quantum corrections to the ground state are thus expected to grow as α → 0, due to the decreasing equatorial barriers, culminating in the extreme case α = 0, where the potential along the equator is flat and barriers vanish. The semiclassical picture thus progressively breaks down as α → 0, and the ground state is correspondingly less well approximated by rotated |*J*, ± *J*⟩ states. For α = 0, they eventually become |*J*, ± 1/2⟩ levels. Concurrently, the **g** matrix deviates increasingly from the maximally axial case. These trends are visible in the sequence of Figures 1d, 1c, and 1a; the deviations of the exact principal *g* values from the maximally axial values provide a good measure for the degree of classicality.

The generic situation is thus that of two isolated, well localized minima, where “well localized” means that the potential is sufficiently steep and the minima sufficiently separated by barriers such that quantum effects are negligible. The ground state is then characterized by a strongly axial doublet with the orientation of the **g** matrix in the direction of the minimum. Whenever the situation is more complicated, because more than two global minima or nearly degenerate local minima or small barrier heights or minimum valleys occur, quantum corrections become increasingly important, to a point where the semiclassical picture breaks down. The reasoning applies analogously to maxima.

Therefore, when the topology of the classical potential *E* ^*cl*^(θ, φ) is such that it exhibits two minima and two maxima, which are isolated and well localized, the system will exhibit strongly axial doublets in both the ground and antiground state, with similar but rotated **g** matrices. In a nutshell, the above paradoxical features are generic for near-classical spins in a ligand field. Instead of characterizing these systems as “low symmetry” it is more appropriate to describe them as “near classical”.

The degree of validity of the semiclassical picture is not easily decided from the qualitative considerations, which seems to call for a quantitative theory. Such a theory would involve sophisticated theoretical machinery^1,40,47^ and satisfactory general results do not appear to exist. However, the degree of validity can always be determined by comparison to the quantum Hamiltonian ([Disp-formula eq1]), which is easily solved numerically—the semiclassical picture does then provide an understanding of the predictions of the quantum Hamiltonian.

An interesting extension of the above is to consider the semiclassical energy *E* ^*sc*^(θ, φ) defined before, due to its connection with the “simple electrostatic model” (SEM) proposed in ref 24 (see also SI). The SEM consists of modeling the lanthanide ion by the 4f charge density ρ^*JJ*^(**r**),^48^ and minimizing the electrostatic energy

$$W^{SEM}\left(\theta ,\varphi \right)=\int {\mathrm{\rho ̃}}^{JJ}\left(\theta ,\varphi ,r\right)V\left(r\right)\;dr$$

for the rotated 4f charge density ${\mathrm{\rho ̃}}^{JJ}\left(\theta ,\varphi ,r\right)$ . The minima of *W* ^*SEM*^(θ, φ) are then taken as the orientation of the main magnetic anisotropy axis.^24^ It turns out that *W* ^*SEM*^(θ, φ) is equal to *E* ^*sc*^(θ, φ).^36^ Therefore, when applying the above semiclassical considerations to *E* ^*sc*^(θ, φ), exactly the same orientations as predicted by the SEM are obtained (and vice versa). Moreover, the semiclassical considerations provide a deep insight into when and why the SEM is applicable.

In summary, this note presents a consistent and intuitive physical picture for the emergence of strongly axial ground doublets in lanthanide clusters with low-symmetry ligand environments, and thereby their SMM behavior. The underlying mechanism is a large total angular momentum *J* of the lanthanides, which drives their behavior toward the classical limit. While this in itself is not surprising, the implications for SMM behavior could be unexpected; the picture resolves a longstanding mystery in the field. Interestingly, the reasoning extends to 3d-based SMMs, effectively obliterating the criterion of strong Ising-type anisotropy. The results presented here do not directly suggest new design rules for SMM synthesis, but they enhance our understanding of the emergence of SMM behavior in lanthanide clusters, which could inform future synthetic efforts.

## Supplementary Material



## Notes

Discussion of subtleties arising in the semiclassical expansion (PDF)
