# Supplementary material for: A Note on Why Low-Symmetry Lanthanide Clusters Can Be Good Single Molecule Magnets
Source: J Am Chem Soc. 2025 Dec 24;148(1):50–4. doi: 10.1021/jacs.5c16415 (PMC12814165; doi:10.1021/jacs.5c16415)
Supplement: Supplementary file 1 [file ja5c16415_si_001.pdf]

Supplementary Information

A Note on Why Low-symmetry Lanthanide Clusters can be good Single Molecule Magnets

Oliver Waldmann

Physikalisches Institut, Universität Freiburg, D-79104 Freiburg, Germany

*Correspondence: oliver.waldmann@physik.uni-freiburg.de

CONTENT

- Supplementary Note S1: Types of semiclassical

<u>Supplementary Note S1: Types of semiclassical</u>

The term "semiclassical" can carry subtly different meanings depending on context, which may be confusing to non-experts. Some comments thus appear appropriate.

The semiclassical approach corresponds to $J \to \infty$. Theoretically it is more useful to think in terms of $1/J \to 0$, as this is the small number. The classical limit then corresponds to $1/J = 0$. Deviations from the classical energy or so-called quantum corrections appear when $1/J$ is small but not zero. The semiclassical approach can thus be described as an expansion in orders of $1/\sqrt{J}$:

$$E^{qm} = E^{cl} + \delta E^{(1)} \frac{1}{\sqrt{J}} + \delta E^{(2)} \frac{1}{\sqrt{J}^2} + \ldots = E^{cl} + \sum_n \delta E^{(n)} J^{-\frac{n}{2}} .$$

While formally unambiguous, the term "semiclassical" can take on subtly different meanings, as different theoretical treatments may yield results that do not precisely match the correct expansion factors $\delta E^{(n)}$, yet are labeled "semiclassical".

For instance, when applying the substitution $\hat{\vec{J}} \to J\vec{n}$, terms of order $1/\sqrt{J}$ and higher can appear (see the example of $\hat{O}_{20}$ below), which however may not coincide with the expansion factors of order $(1/J)^{n/2}$ in a full expansion. Typically, also zero-point energies due to quantum fluctuations, which can be calculated, e.g., using bosonization techniques (such as Holstein-Primakoff bosons), need to be considered in addition.

This note alone presents three slightly distinct notions of "semiclassical", which can be interpreted as different substitution methods for converting operators into their (semi)classical versions:

#1: Substitution $\hat{\vec{J}} \to J\vec{n}$:

A good (since simple) example is the operator $\hat{O}_{20} = 3\hat{J}_z^2 - J(J+1)$. The substitution $\hat{\vec{J}} \to J\vec{n}$ leads to $O_{20} = 3J^2 \cos^2\theta - J(J+1)$ or

$$E(\theta,\varphi)/J^2 = \left(3\cos^2\theta - 1\right) - \frac{1}{J}.$$

A correction to the classical limit of order $1/J$ appears (last terms on the r.h.s.). However, the contribution $-J^{-1}$ is not the correct expansion factor $\delta E^{(2)} J^{-1}$ in the full expansion, as a full calculation would reveal (additional contributions arise from zero-point fluctuations; the calculation and result are beyond the scope of this note). This substitution method is thus - in general - incomplete and does not yield the exact semiclassical expansion.

#2: Substitution $\hat{Y}_{kq} \to J^k Y_{kq}$:

This approach actually yields precisely the classical limit, and is thus not "semiclassical" in the strict, above sense.

#3: Substitution $\hat{Y}_{kq} \to \sqrt{\frac{4\pi}{2k+1}} \langle JJ | \hat{Y}_{k0} | JJ \rangle Y_{kq}$:

This approach is motivated by the SEM, for which the energy is calculated as

$$E^{SEM}(\theta,\varphi) = \sum_{kq} C_{kq} \sqrt{\frac{4\pi}{2k+1}} \langle JJ | \hat{Y}_{k0} | JJ \rangle Y_{kq}(\theta,\varphi),$$

where $\langle JJ | \hat{Y}_{k0} | JJ \rangle$ is the matrix element of $\hat{Y}_{k0}$ in the state $|J,J\rangle$.[1,2] This result can equivalently be generated from the Hamiltonian $\hat{H} = \sum_{kq} C_{kq} \hat{Y}_{kq}$ by the substitution $\hat{Y}_{kq} \to \sqrt{4\pi/(2k+1)} \langle JJ | \hat{Y}_{k0} | JJ \rangle Y_{kq}(\theta,\varphi)$. For the operators $\hat{Y}_{k0}$, i.e., for $q = 0$, this happens to give the exact same results as the substitution method #1, since the pre-factor happens to precisely account for the $1/J$ factor appearing in the substitution $\hat{\vec{J}} \to J\vec{n}$.[1] However, this is not necessarily so for $q \neq 0$, and approaches #1 and #3 are thus not equivalent. Moreover, similar to method #1, method #3 is also incomplete, and does not yield the correct semiclassical expansion.

Using the energy of any semiclassical method which creates $1/\sqrt{J}$ corrections can be considered an improvement over using the classical energy, and quantitatively this may indeed be the case. However, this is not guaranteed to be so, as it is not derived from a systematic expansion, and the energy may even be less accurate.

As a summary of this supplemental note, the point to be aware of is that different semiclassical methods exist, each giving slightly different results for the energy. They may agree slightly better with the exact result, but they may also be less accurate. Generally, when the classical and the various semiclassical results are not close to each other, the validity or applicability of the semiclassical approach should be put in question.
